# Genetic diversity, population structure and subdivision of local Balkan pig breeds in Austria, Croatia, Serbia and Bosnia-Herzegovina and its practical value in conservation programs

**DOI:** 10.1186/1297-9686-44-5

**Published:** 2012-03-01

**Authors:** Thomas Druml, Kresimir Salajpal, Maria Dikic, Miroslav Urosevic, Gertrud Grilz-Seger, Roswitha Baumung

**Affiliations:** 1 BOKU University of Natural Resources and Life Sciences, Gregor Mendel Str. 33, 1180 Vienna, Austria; 2 Faculty of Agriculture, Department of Animal Science, University of Zagreb, Svetosimunska c25, 10000 Zagreb, Croatia; 3 Institute of Food Technology, University of Novi Sad, Bulevar Cara Lazara 1, Novi Sad, Serbia; 4 Pöckau 41, 9601 Arnoldstein, Austria

## Abstract

**Background:**

At present the Croatian Turopolje pig population comprises about 157 breeding animals. In Austria, 324 Turopolje pigs originating from six Croatian founder animals are registered. Multiple bottlenecks have occurred in this population, one major one rather recently and several more older and moderate ones. In addition, it has been subdivided into three subpopulations, one in Austria and two in Croatia, with restricted gene flow. These specificities explain the delicate situation of this endangered Croatian lard-type pig breed.

**Methods:**

In order to identify candidate breeding animals or gene pools for future conservation breeding programs, we studied the genetic diversity and population structure of this breed using microsatellite data from 197 individuals belonging to five different breeds.

**Results:**

The genetic diversity of the Turopolje pig is dramatically low with observed heterozygosities values ranging from 0.38 to 0.57. Split into three populations since 1994, two genetic clusters could be identified: one highly conserved Croatian gene pool in Turopoljski Lug and the"Posavina" gene pool mainly present in the Austrian population. The second Croatian subpopulation in Lonjsko Polje in the Posavina region shows a constant gene flow from the Turopoljski Lug animals.

**Conclusions:**

One practical conclusion is that it is necessary to develop a "Posavina" boar line to preserve the "Posavina" gene pool and constitute a corresponding population in Croatia. Animals of the highly inbred herd in Turopoljski Lug should not be crossed with animals of other populations since they represent a specific phenotype-genotype combination. However to increase the genetic diversity of this herd, a program to optimize its sex ratio should be carried out, as was done in the Austrian population where the level of heterozygosity has remained moderate despite its heavy bottleneck in 1994.

## Background

Many studies on the diversity of various pig breeds have been reported in the literature in recent years. As early as the 1990's, several research groups have worked on harmonizing data from these various genetic studies in multiple pig populations from Europe and China (PiGMaP [1], PigBioDiv1 and 2 [2-4]). For instance, the PigBioDiv projects include, among other data, around 50 European and 50 Chinese pig populations and a set of 50 microsatellite markers to study the pig genetic diversity in the main breeding and production areas. The major objective of these projects was to demonstrate how relevant analyses in overlapping countries can be carried out to study genetic diversity. Other approaches have also opened up new prospects in this field of research. In 2010, Boitard et al. [5] have reported a large-scale analysis on the genetic diversity and population structure of French and Spanish pig breeds based on 5791 animals from 22 commercial populations. Besides from assigning individuals to populations, this work also investigated how the relatedness between individuals and the differences in sampling times affected statistical analyses.

Larson et al. [6,7] have studied the domestication process of European and Asian wild boar and domestic pigs based on mitochondrial D-loop sequence analyses in a wide range of pig breeds. These authors detected two separate haplotype groups in Europe: a Central European (D1) group and a Mediterranean group, which fits with previous reports on pig domestication [8,9]. The Balkan pig breeds such as the Turopolje and Mangalica breeds are generally assumed to originate from the Mediterranean type, but to date neither their genetic diversity nor their evolutionary status in the haplotype networks have been analysed.

In this study, our aim was to illustrate the diversity and genetic relationships of local pig breeds originating from Croatia, Serbia, Hungary and Bosnia-Herzegovina. The Croatian Turopolje breed has undergone a complicated recent breed history. In 1991, the last Turopolje pigs of the Posavina region in Croatia were collected to establish a nucleus herd for further conservation programs. However, in the autumn of the same year the Yugoslavian wars started and put a stop to this project initiated by the SAVE Foundation and EURONATURE. Six animals were rescued and placed in the Zagreb zoo. Four of these animals, together with two boars imported from the Posavina region in 2001, constituted the founder population for the Turopolje breed in Austria, which comprises at present 324 herdbook animals. A recently conducted pedigree analysis showed the high levels of inbreeding (F = 12%, mean pedigree depth of four generations) and the poor variability of the Austrian gene pool [10,11]. After the end of the Croatian war in 1994, the SAVE Foundation and the Croatian state started to work on the project again within the context of the Lonjsko Polje National park. In order to build up a new nucleus herd of the Croatian pig breed, additional breeding animals from the Turopolje nucleus herd in Turopoljski Lug near Zagreb were used. This herd represents the second genetic resource for this local and autochthonous Croatian breed and has an uninterrupted breeding history dating back to the 19^th ^century. At present, this subpopulation comprises 90 breeding animals and over the last 60 years, it has not undergone any immigration of pigs from other origins [12].

**Figure 1:**
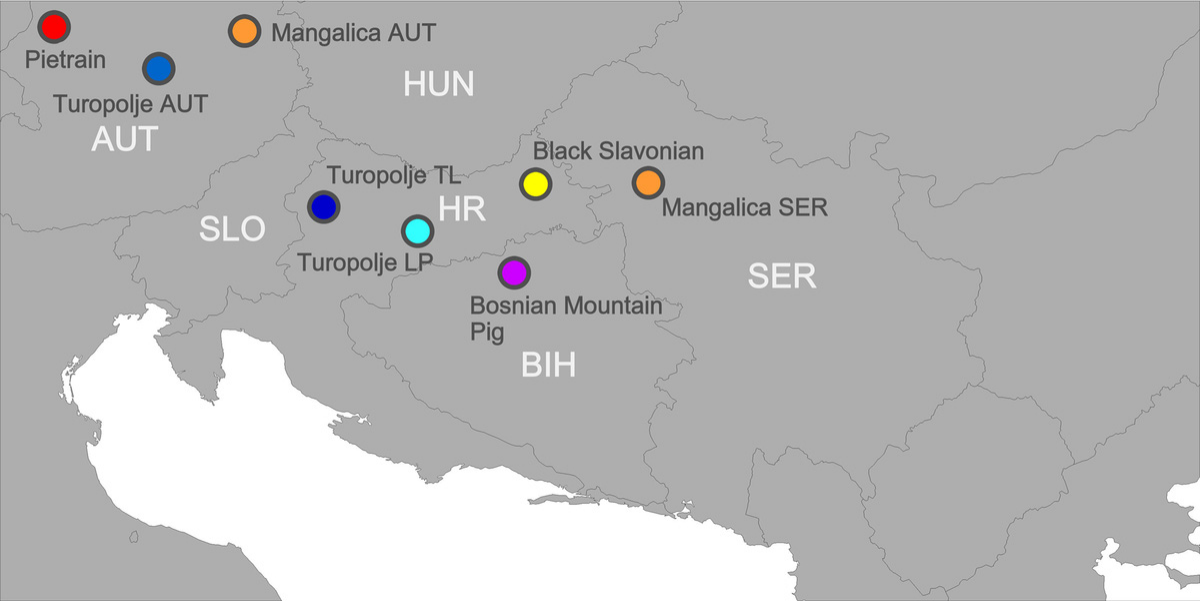
**Map of the Balkan countries and sampling areas**. Turopolje TL = Turopolje herd in Turopoljski Lug, Turopolje LP = Turopolje herd in the national park Lonjsko Polje, Turopolje AUT = Austrian Turopolje population, Black Slavonian population, Mangalica SER = Serbian Mangalica population from the national park Zasavica, Bosnian mountain pig = Bosnian mountain pig in the district of Modrica, Mangalica AUT = Austrian Mangalica population, Pietrain = Austrian Pietrain

Because in the Austrian population the level of inbreeding is very high, our aim was to genetically characterize sub-populations and individuals from the Croatian pig breed in Turopoljski Lug, which could be used for future breeding programs in Austria. We studied the genetic diversity within the three Turopolje populations and their genetic relationships. In order to take into consideration, the influence that the instable demographic and political situation during and after the Croatian war may have had on the breeding of Turopolje pigs in the Save delta, we included animals from other local pig breeds localized next to this area i.e. the Black Slavonian or Fajferica, a local lard-type breed of the eastern part of Croatia, the Bosnian mountain pig, a local mixed population on the Bosnian side of the Save river and Austrian and Serbian Mangalica pigs, which originate from the border region of Serbia and Croatia (Figure 1).

## Methods

### Populations

Hair samples were collected on field trips in Croatia, Serbia and Bosnia Herzegovina in 2009 and 2010. Samples of extracted DNA for parentage controls were also used, which included all unrelated Austrian pig individuals and part of the Croatian individuals sampled in 2003 and five Turopolje pigs sampled in 1998. A total of 177 pigs were sampled i.e. 93 Turopolje pigs (29 from the herd in Turopoljski Lug (TL), 39 from the herd of the Lonjsko Polje national park (LP) and 25 from the Turopolje population of Austria (AUT), 41 Black Slavonian pigs, 23 Mangalica pigs from the Austrian (AUT) herdbook (mainly imported animals from Hungary), 11 Serbian Black Mangalica pigs from the national Zasavica park (SER), and nine pigs from a valley in Bosnia and Herzegovina, Republika Srpska. Since the small-sized Bosnian samples may cause distortion in the phylogenetic studies, they were excluded but the Bosnian mountain pigs were retained for the analysis of gene diversity and population structure. Samples of 20 unrelated Austrian Pietrain pigs were also included, as outgroup individuals since this breed is quite distant from the lard-type breeds of the Balkan countries both in terms of production traits mean values and genetic units. Finally, the DNA from 197 animals from eight populations and five breeds was used. The experimental data in this paper were obtained according to the legislative regulations regarding experimentals research on animals in Austria, Croatia, Serbia and Bosnia i Herzegovina.

### Markers

All animals were genotyped for 25 microsatellite markers belonging to the panel recommended by the ISAG-FAO Advisory Committee for genetic distance studies [13]. Twelve of these microsatellites are currently used for parentage controls in Austria for the Turopolje and Mangalica breeds. However, microsatellites S005, S0090 and SW24 were discarded because of the poor reproducibility of their allele determination. PCR-amplified microsatellite markers were analysed with an ABI PRISM 310 genetic analyzer and Genescan. In some cases, the quality of the extracted DNA was to low for further analyses due to inadequate storage or transport i.e. samples from four Austrian Turopolje, six Austrian Mangalicas, three Austrian Pietrain, two Croatian Turopolje and one Croatian Black Slavonian pigs had to be excluded from the analysis. Thus 181 animals were finally included in the statistical analysis.

### Analyses

#### Within-breed diversity

Allele frequency, number of alleles, observed heterozygosity (HO) and gene diversity (HE) were calculated across different loci and populations using the GENETIX 4.05.2 software package [14]. Mean number of alleles (MNA) and rarefacted mean numbers of alleles (RMNA) were calculated using MOLKIN 2.0 [15]. Tests for deviations from Hardy-Weinberg equilibrium (HWE) were performed across all loci by GENEPOP 3.4 [16] applying the exact test with default settings of the Markov chain Monte Carlo methodology. Wright's F_IS _was calculated according to Weir and Cockerham [17] using GENETIX 4.05.2 [14]. To account for bottlenecks in the populations, the software package BOTTLENECK 1.2.02. [18] was applied with a two-phased model of mutation (TPM), consisting of 95% of strict one-step mutation steps (SMM) and 5% of multi-step changes (IAM). Allele frequency distribution (mode shift indicator) was used to illustrate the bottlenecks which were tested simultaneously by the Wilcoxon test [19].

#### Genetic distances and trees

Molecular genetic relationships among populations were derived using Wright's F_ST _[17] and the Reynolds distance [20]. Individual relationships were calculated using Cavalli-Sforza's D_k _distance [21]. Among the many genetic distance measures available, the Reynolds distance is considered to be the best to analyze populations that have recently diverged and neighbour joining (NJ) is the preferred technique for tree building when the populations under study differ in their effective population size [22,23].

All genetic distances were calculated using the software package POPULATIONS 1.2.28 [24]. The stability of each node for the population level distances was evaluated with 1000 bootstraps samples. A principal component analysis of the individual D_k _matrix was performed using the SAS^® ^software [25].

#### Population structure

Population subdivisions and genetic differentiation can be tested by several clustering procedures. However, the algorithm of Pritchard et al. [26], implemented in the software STRUCTURE 2.1 has become a frequently used application in genetic diversity studies. The program can estimate "hidden structures» i.e. the number of different clusters (K partitions) obtained without using any prior information about individual membership (population and/or breed). Moreover, for each individual, the program is able to determine the proportion of genes originating from the K potential clusters. Based on the Markov chain Monte Carlo (MCMC) method, the STRUCTURE algorithm uses a model-based approach, under the hypothesis that the loci are in Hardy-Weinberg equilibrium and in linkage equilibrium within clusters, to define the natural logarithm of the probability that a given genotype belongs to the assumed K clusters. The program was run 10 times for each K, starting from K = 2 to K = 11. The results presented in this study are based on 10^6 ^iterations following a burn-in period of 10^5 ^iterations, applying an admixture model, correlated frequencies model and the parameter of individual admixture alpha set to the same values for all clusters and with a uniform prior. The resulting individual genotype membership coefficients were displayed using DISTRUCT [27].

## Results

### Within-breed diversity

The total number of alleles found for the 22 microsatellite markers analyzed on the eight pig populations amounted to 171 with a mean number of alleles per locus of 7.7, ranging between five and 11 alleles. The observed heterozygosity level ranged from 0.15 for loci *S0227 *and *SW951 *to 0.83 for loci *SW939 *and *S0097*. A significant deviation from HWE was observed for loci *SW951, S0387 *and *SW2410 *and although the existence of null alleles for these three loci was not demonstrated, they were excluded from further analyses (compare additional file 1).

Data on the genetic diversity within the eight pig populations studied are presented in Table 1. Observed heterozygosity and gene diversity ranged from 0.38 and 0.37 (Turopolje TL) to 0.64 and 0.66 (Pietrain), respectively. Allele richness was lowest in the Turopolje TL breed (MNA = 3.29) and highest in the Black Slavonian breed (MNA = 5.37). However, since the mean number of alleles is influenced by sample size, it was adjusted for sample size (22 genes), but allele richness remained lowest in the Turopolje TL breed (RMNA = 2.31) while it was highest in the Pietrain breed (4.15).

**Table 1:** Genetic diversity within the 8 pig populations studied

Population	N	HO	HE	MNA	RMNA	Fis	negative Fis sign
Bosnian mountain pig	9	0.62	0.58	4.16	3.89	0.01	NS

Mangalica AUT	17	0.49	0.54	3.8	3.37	0.01	NS

Mangalica SER	11	0.58	0.54	3.94	3.57	-0.06	*

Black Slavonian	40	0.59	0.64	5.37	3.98	0.01	NS

Pietrain	17	0.64	0.66	5.02	4.15	0.01	NS

Turopolje AUT	21	0.48	0.54	4.53	3.55	0.07	NS

Turopolje Lonjsko Polje	29	0.57	0.55	4.45	3.34	-0.04	NS

Turopolje Turopoljski Lug	37	0.38	0.37	3.29	2.31	-0.11	*

**TOTAL**	**181**	**0.54**	**0.66**	**7.74**	**4.45**		

The results are based on 19 microsatellite loci from 181 animals; sample size (N), observed heterozygosity (HO), gene diversity (HE), mean number of alleles (MNA), the rarefacted mean number of alleles on a sample with 22 genes (RMNA), Wright's F_IS._and 95% confidence interval of F_IS._, * indicates a significant difference of F_IS _smaller than zero (p < 0.05)

Negative F_IS _coefficients, with a 95% confidence interval ranging from -0.20 to -0.04, were estimated for the Serbian Mangalica and Turopolje TL breeds, suggesting an excess of heterozygotes due to non-random mating. In all other populations F_IS _values were not significantly different from zero. Overall, low average diversity values (heterozygosity and allele richness) were observed in all three Turopolje populations (Turopolje TL, Turopolje LP, Turopolje AUT) illustrating the risk status of this breed.

Applying the Wilcoxon sign-rank test [19] for gene diversity excess or gene diversity deficit, a significant genetic bottleneck was revealed only for the Austrian Turopolje population (*p *= 0.013, two sided test *p *= 0.027).

### Genetic distances and trees

**Figure 2:**
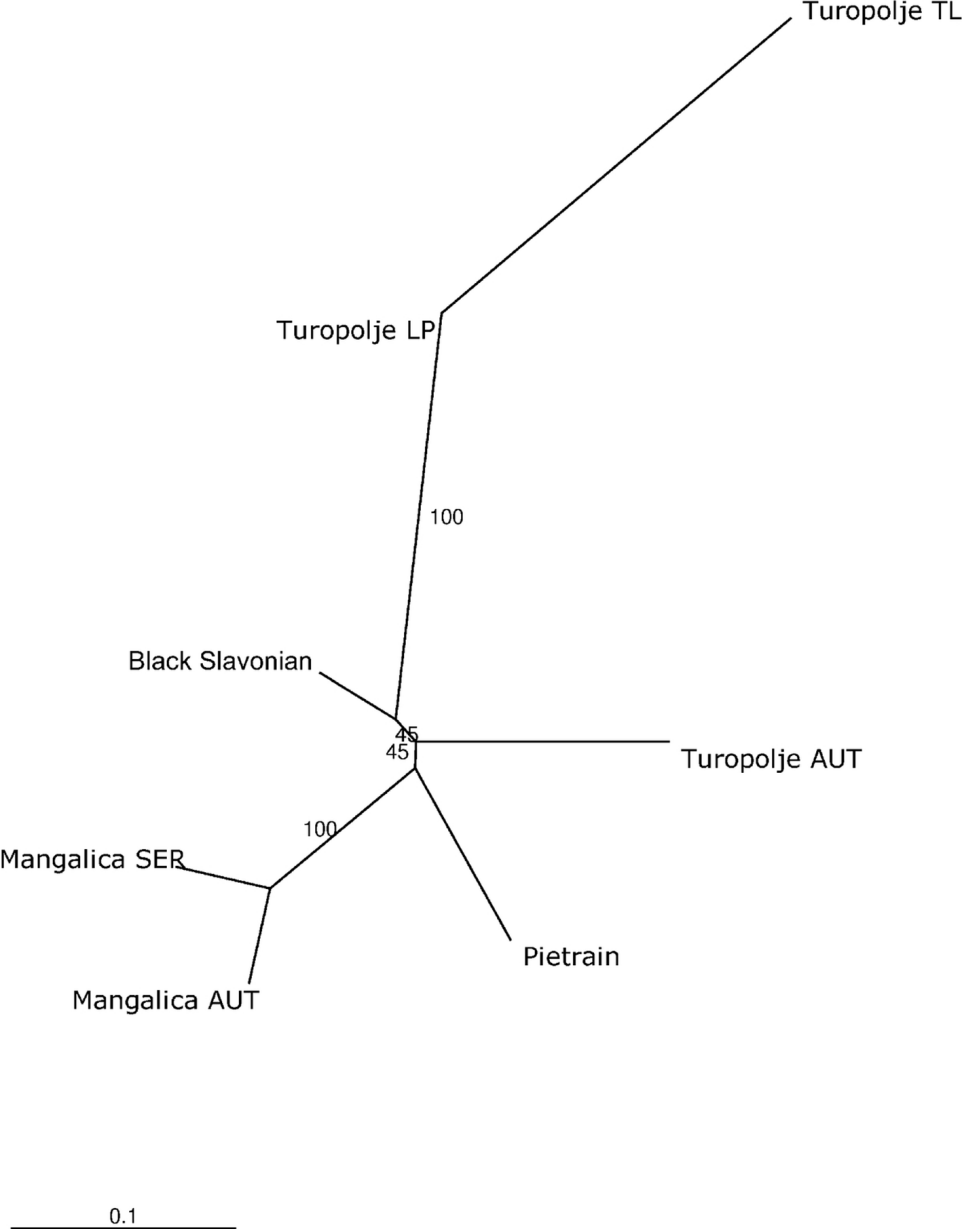
**Dendrogram on the population level**. Unrooted neighbor joining tree for seven populations (excluding Bosnian mountain pig) from four different breeds based on the Reynold's distance

The largest differentiation expressed by F_ST _values was observed between the Turopolje TL and Turopolje AUT populations (F_ST _= 0.26). Lower differentiation levels ranging from F_ST _= 0.05 to F_ST _= 0.09, were observed between the Black Slavonian and Mangalica breeds (AUT and SER), Bosnian mountain, and the Austrian Turopolje pig populations and between the Turopolje TL Turopolje LP populations (for further information see additional files 2 and 3). Genetic distances between populations are shown in Figure 2, where the Turopolje TL population occupies the most distant position within the dendrogram.

**Figure 3:**
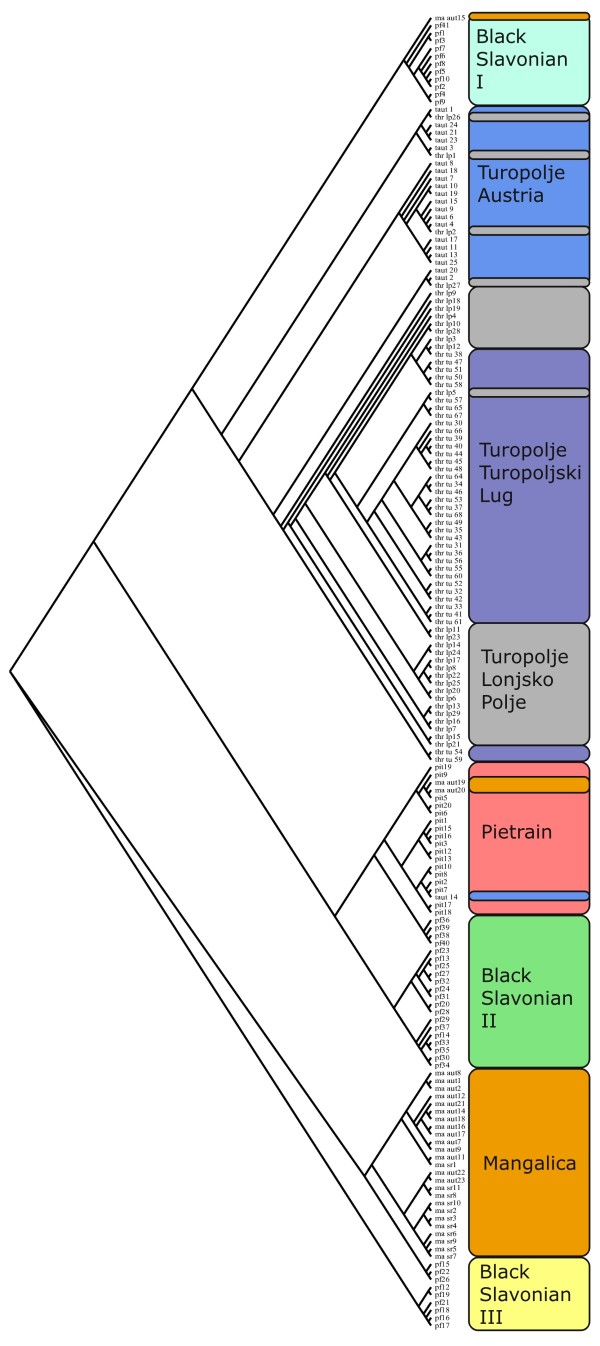
**Individual neighbor joining tree**. Dendrogram of 172 individuals from four different pig breeds (seven populations) derived from the D_K _using neighbor joining clustering. Color bars correspond to the population: grey = Turopolje Lonjsko Polje, blue = Turopolje Austria, purple = Turopolje Turopoljski Lug, light blue = Black Slavonian I, green = Black Slavonian II, yellow = Black Slavonian III, red = Pietrain Austria, orange = Mangalica Austria and Serbia

Individual clustering is presented in the neighbour joining tree based on the D_k _distance in Figure 3. The three Turopolje populations form a monophyletic cluster concordant with their recent breed history. On the same branch, a Black Slavonian (I) cluster is identified representing the herd of a single breeder near Vinkovci (HR). Both Mangalica populations are monophyletic whereas a paraphyletic cluster is observed for the Black Slavonian population (III) representing a herd of another breeder in Durici (HR). the Austrian populations, we identified three animals, one Turopolje and two Mangalicas, clustering with the Pietrain breed.

### Population structure

**Figure 4:**
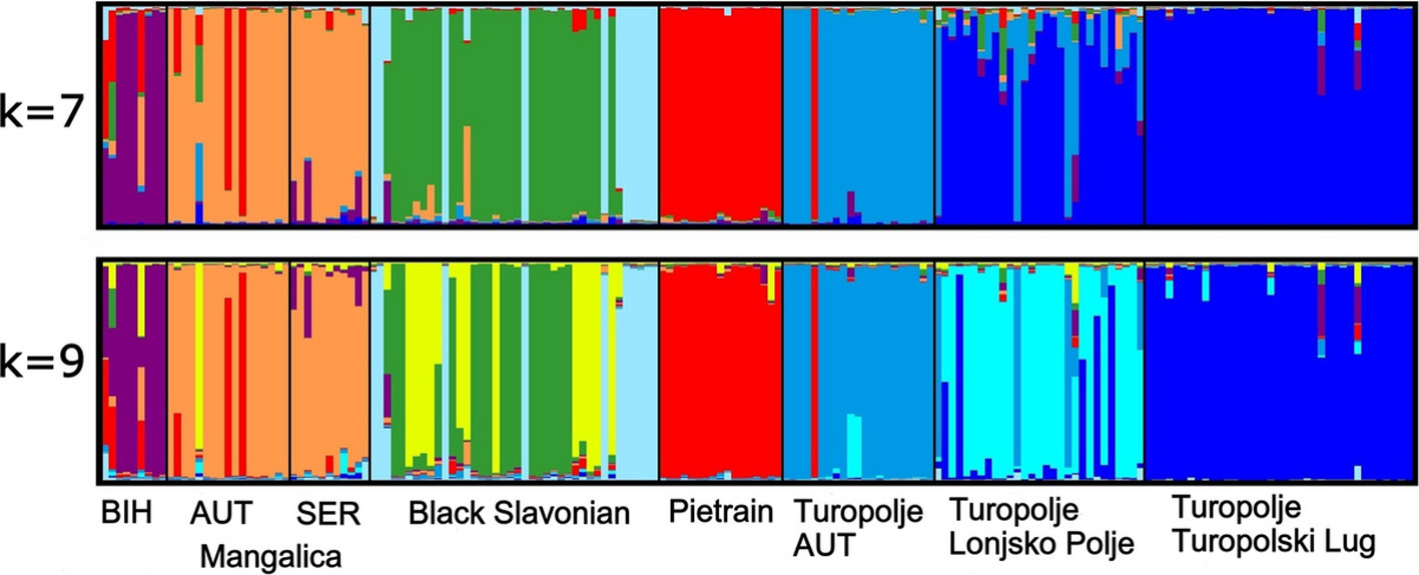
**Bayesian clustering (181 animals) using 19 markers**. The clustering was obtained from STRUCTURE, for a model with admixture and correlated allele frequencies between groups; each pig is represented by a single vertical line broken into K colour segments, with lengths proportional to the estimated membership of the inferred cluster; only graphical presentations with the best fits towards data (K = 7 and K = 9) are shown; BIH = Bosnian Mountain pig; Mangalica AUT = Austrian Mangalica population; Mangalica SER = Serbian Mangalica

To measure the population structure and degree of admixture, we applied the STRUCTURE algorithm. All runs from K = 2 to K = 9 showed a pattern allowing a meaningful interpretation. However, the highest log likelihood score was obtained for K = 9. The log likelihood values for K = 7, forming a semi-plateau, showed little variability among 10 independent runs (compare additional file 4). Thus, the status K = 7 corresponds to the picture of breed formation, whereas the status K = 9 represents a higher resolution of the actual state of the composition of the local Croatian breeds (Figure 4). The population of the Lonjsko Polje national park is assigned to a separate gene pool and the Black Slavonian breed is split into three different pools represented by at least three different breeding herds. The Bosnian mountain population forms a separate pool and the Mangalica populations cluster together probably because of a common genetic background since the Hungarian imports played an important role in both populations. The same three animals (two Mangalica individuals and one Austrian Turopolje) as observed in Figure 3, carried a Pietrain genotype.

## Discussion

The genetic diversity within the different Balkan pig breeds studied is low compared to that of conventional breeds playing a key role in global meat production. Numerous studies have reported observed heterozygosity values for different populations of the Landrace, Large White, Pietrain and Duroc breeds with values ranging from 0.67 to 0.50 [1,3-5,23]. The economically less important local European breeds often show reduced census and effective population sizes [28,29]. Therefore their diversity measured by genetic markers is significantly decreased and ranges from HO = 0.35 to HO = 0.58 [1,23]. The population history of commercial European pig breeds such as Large White or Landrace is characterized by several events of introgression through Asian genotypes during the 19th century. This larger and heterogeneous founder gene pool as well as controlled breeding programs and the increase of population sizes throughout the 20^th ^century may be responsible for the higher level of diversity within these breeds. The populations of Balkan pig breeds analyzed in this study show lower levels of heterozygosity, except the Bosnian mountain pig, where phenotypically crossbred individuals could be identified. Considering only the Turopolje breed, it is clear that the nucleus herd in Turopoljski Lug lacks genetic variability (HO = 0.38; MNA = 3.29; RMNA = 2.31), although its F_IS _value is significantly lower than zero, suggesting that there are more heterozygous animals than expected. This contradiction may be explained by the breeding history of this population. The nucleus herd is managed by a farmers' collective and at present, although the population is split into two herds, the breeders are using only one or in the past two boars for reproduction. This mating strategy results in genetic and phenotypic uniformity and high levels of inbreeding within the boar lines. The merging of such boar lines is comparable to cross-breeding, which is documented here by the negative F_IS _value. Situations in the Austrian Turopolje herd with high heterozygosity values reaching 0.48 and in the nucleus herd in Turopoljski Lug differ completely. This is due to of the fact that a narrow sex ratio (2.7 females per male) has been maintained during the last 17 years.

The Turopolje herd of the national park Lonjsko Polje, genealogically a metapopulation of Turopolje TL and Turopolje AUT, shows the highest levels of genetic diversity (HO = 0.57), which is the consequence of using Turopoljski Lug animals since 1994.

Generally the Balkan pig populations are well differentiated as demonstrated by the mean F_ST _value of 0.24, which is comparable to the results reported by SanCristobal et al. [3] (mean F_ST _= 0.21) and Fabuel et al. [22] (mean F_ST _= 0.14 - 0.15). The Turopolje island population in Turopoljski Lug is highly differentiated compared to all the other studied breeds (mean F_ST _= 0.21). The highest F_ST _value is obtained within the Turopolje breed surprisingly with a coefficient of 0.26 between Turopolje TL and Turopolje AUT. The Black Slavonian is moderately differentiated (mean F_ST _value of 0.10) and overlaps with the Austrian Turopolje and the Mangalica breeds, a result also observed in the individual-based phylogenetic tree (Figure 3). The results obtained from STRUCTURE and the individual genetic distances suggest that a moderate gene flow has occurred between the Black Slavonian breed and the Turopolje population of the Posavina. More precise data on this genetic relationship could be obtained by analyzing mitochondrial DNA.

## Conclusions

The aim of this study was to identify possible candidate populations and individuals from the Croatian local pig breeds, which could be used to increase the gene pool of Austrian Turopolje pigs. Considering the results, genetic distances and population structure, three populations could be used in the Austria conservation program: the mother population in Lonjsko Polje, the second Croatian nucleus herd in Turopoljski Lug, and a breeding group from the Black Slavonian breed (Figure 3: Black Slavonian I). However, the situation for the Turopolje pig breed remains very serious. In Croatia, only about 157 breeding animals (in two populations) are left, and the Austrian population (at present, 324 breeding animals) is isolated due to the import regulations of the European Union. The results of this study suggest that the Turopolje herd in Lonjsko Polje is a metapopulation between the herd of Turopoljski Lug and the Austrian population. At present, the gene pool of the Austrian herd originates from the old "Posavina" gene pool and its size is continuously decreasing in Lonjsko Polje due to animals immigrating from Turopoljski Lug. Only a few animals from this herd still belong to the "Posavina" cluster and it is important to develop a boar line out of this genetic cluster in order to preserve this gene pool for the future. The herd in Turopoljski Lug is a product of a strict consolidation breeding program. The phenotypically distinct animals are highly inbred and the genetic diversity is low, but no sign of inbreeding depression is observed for the time being. Thus, establishing one or two additional boar lines could help to increase genetic diversity in the long term.

## Competing interests

The authors declare that they have no competing interests.

## Authors' contributions

TD participated in the design of the study, organized and led the field work, performed the statistical analysis and drafted the manuscript. KS and MD carried out the DNA extraction. MU and GGS participated in the field work and drafted the manuscript. RB conceived of the study, and participated in its design and coordination. All authors read and approved the final manuscript.

## Supplementary Material

Additional file 1 **List of markers used for this study**. Alleles, number of alleles, observed heterozygosity (HO) and polymorphic information content of the 22 markers typed in this study; markers with a * were excluded from further analyses due to deviations from the Hardy-Weinberg equilibrium. Click here for file

Additional file 2 **Population differentiation based on F_ST _estimates among eight pig populations**. Values are inferred from 19 microsatellite markers; on the diagonal F_IS _values are shown in bold. Click here for file

Additional file 3 **Principal component analysis**. Description: Plot of the first three principal component axes based on individual genetic distance D_K _matrices; ellipsoids contain 75% of the animals; 1 red = Mangalica AUT, 2 orange = Mangalica SER, 3 green = Black Slavonian, 4 blue = Pietrain, 5 black = Turopolje AUT, 6 black = Turopolje Lonjsko Polje, 7 black = Turopolje Turopoljski Lug. Click here for file

Additional file 4 **Distribution of ln(X|K) from ten iterations ranging from K = 2 to K = 10**. Values of log likelihood of the multilocus genotypic data, ln(X|K), as a function of the number of clusters, K (ten runs); the largest values of ln(X|K) are presented with black dots. Click here for file
