# Supplementary material for: Genetic diversity, population structure and subdivision of local Balkan pig breeds in Austria, Croatia, Serbia and Bosnia-Herzegovina and its practical value in conservation programs
Source: Genet Sel Evol. 2012 Mar 1;44(1):5. doi: 10.1186/1297-9686-44-5 (PMC3311151; doi:10.1186/1297-9686-44-5)
Supplement: Additional file 1 — List of markers used for this study. Alleles, number of alleles, observed heterozygosity (HO) and polymorphic information content of the 22 markers typed in this study; markers with a * were excluded from further analyses due to deviations from the Hardy-Weinberg equilibrium. [file 1297-9686-44-5-S1.DOC]

| **Marker** | **Alleles (bp)** | **NA** | **HO** | **PIC** |
| --- | --- | --- | --- | --- |
| S0026 | 92, 94, 96, 98, 102 | 5 | 0.51 | 0.46 |
| S0097 | 203, 205, 213, 215, 229, 231, 233, 235, 237, 239, 241 | 11 | 0.83 | 0.72 |
| S0101 | 190, 194, 198, 202, 204, 206, 208, 210, 212 | 9 | 0.76 | 0.60 |
| S0155 | 150, 156, 158, 160, 162, 164, 166 | 7 | 0.76 | 0.59 |
| S0178 | 98, 106, 108, 110, 112, 114, 116, 118, 120, 122, 124 | 11 | 0.81 | 0.66 |
| S0226 | 175, 177, 183, 189, 191, 195, 197, 205 | 8 | 0.69 | 0.58 |
| S0227 | 228, 238, 240, 250, 252 | 5 | 0.15 | 0.14 |
| S0228 | 218, 222, 224, 228, 232, 234, 238 | 7 | 0.39 | 0.32 |
| S0355 | 245, 249, 251, 259, 273 | 5 | 0.53 | 0.43 |
| S0386* | 166, 168, 170, 174, 176, 178, 180, 182, 184 | *9* | *0.66* | *0.48* |
| SW0143 | 148, 150, 154, 156, 158, 160, 162, 164 | 8 | 0.55 | 0.47 |
| SW0830 | 180, 182, 184, 186, 188, 190, 192 | 7 | 0.64 | 0.49 |
| SW1067_01 | 151, 157, 159, 161, 163, 165, 167, 169, 171, 173, 175 | 11 | 0.81 | 0.70 |
| SW2008 | 90, 96, 98, 100, 102, 104 | 6 | 0.68 | 0.48 |
| SW240 | 94, 96, 98, 106, 108, 110, 112, 114, 116 | 9 | 0.82 | 0.69 |
| SW2410* | 106, 108, 110, 112, 114, 116, 118, 120, 122, 124 | *8* | *0.71* | *0.61* |
| SW72 | 96, 104, 106, 108, 112, 114, 116 | 7 | 0.68 | 0.55 |
| SW857 | 138, 143, 147, 149, 151, 153, 155 | 7 | 0.64 | 0.46 |
| SW911 | 154, 158, 160, 162, 164, 166, 170 | 7 | 0.71 | 0.49 |
| SW936 | 91, 93, 95, 97, 101, 105, 107, 109, 113 | 9 | 0.83 | 0.68 |
| SW951* | 120, 122, 128, 130, 132 | *5* | *0.15* | *0.14* |
| Swr1941 | 201, 207, 209, 211, 213, 215, 219, 221 | 8 | 0.77 | 0.62 |
