# Supplementary material for: Genetic diversity, population structure and subdivision of local Balkan pig breeds in Austria, Croatia, Serbia and Bosnia-Herzegovina and its practical value in conservation programs
Source: Genet Sel Evol. 2012 Mar 1;44(1):5. doi: 10.1186/1297-9686-44-5 (PMC3311151; doi:10.1186/1297-9686-44-5)
Supplement: Additional file 2 — Population differentiation based on FST estimates among eight pig populations. Values are inferred from 19 microsatellite markers; on the diagonal FIS values are shown in bold. [file 1297-9686-44-5-S2.DOC]

|  |  | 1 | 2 | 3 | 4 | 5 | 6 | 7 | 8 |
| --- | --- | --- | --- | --- | --- | --- | --- | --- | --- |
| Bosnian mountain pig | 1 | **0.01** |  |  |  |  |  |  |  |
| Mangalica AUT | 2 | 0.11 | **0.09** |  |  |  |  |  |  |
| Mangalica SER | 3 | 0.10 | 0.06 | **-0.06** |  |  |  |  |  |
| Black Slavonian | 4 | 0.05 | 0.09 | 0.08 | **0.01** |  |  |  |  |
| Pietrain | 5 | 0.08 | 0.12 | 0.12 | 0.08 | **0.01** |  |  |  |
| Turopolje AUT | 6 | 0.10 | 0.13 | 0.13 | 0.08 | 0.12 | **0.07** |  |  |
| Turopolje Lonjsko Polje | 7 | 0.09 | 0.14 | 0.12 | 0.10 | 0.12 | 0.12 | **-0.04** |  |
| Turopolje Turopoljski Lug | 8 | 0.17 | 0.25 | 0.21 | 0.20 | 0.21 | 0.26 | 0.08 | **-0.10** |
