# Supplementary figures and images for: Genetic diversity, population structure and subdivision of local Balkan pig breeds in Austria, Croatia, Serbia and Bosnia-Herzegovina and its practical value in conservation programs
Source: Genet Sel Evol. 2012 Mar 1;44(1):5. doi: 10.1186/1297-9686-44-5 (PMC3311151; doi:10.1186/1297-9686-44-5)

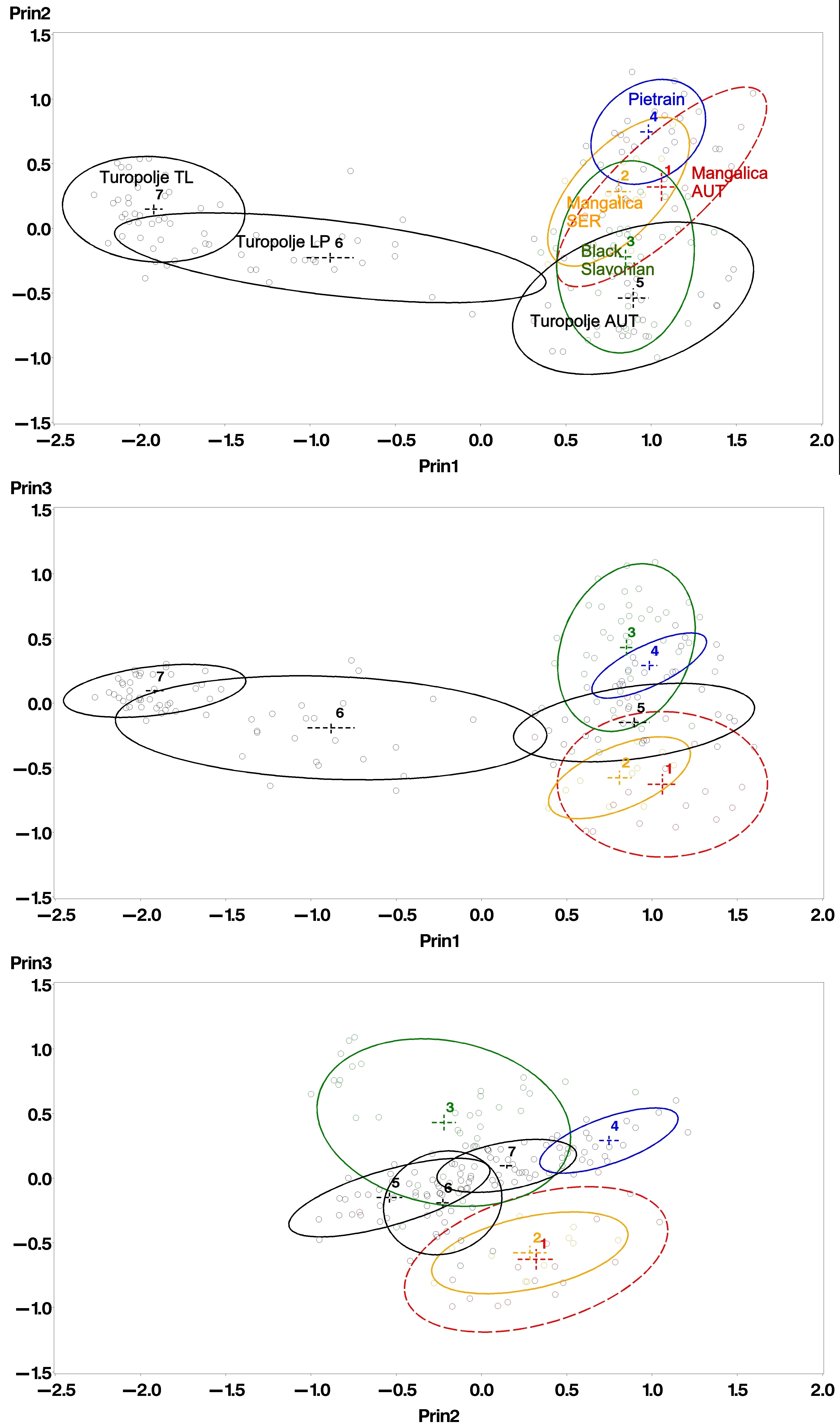

Supplement: Additional file 3 — Principal component analysis. Description: Plot of the first three principal component axes based on individual genetic distance DK matrices; ellipsoids contain 75% of the animals; 1 red = Mangalica AUT, 2 orange = Mangalica SER, 3 green = Black Slavonian, 4 blue = Pietrain, 5 black = Turopolje AUT, 6 black = Turopolje Lonjsko Polje, 7 black = Turopolje Turopoljski Lug. [file 1297-9686-44-5-S3.JPEG]

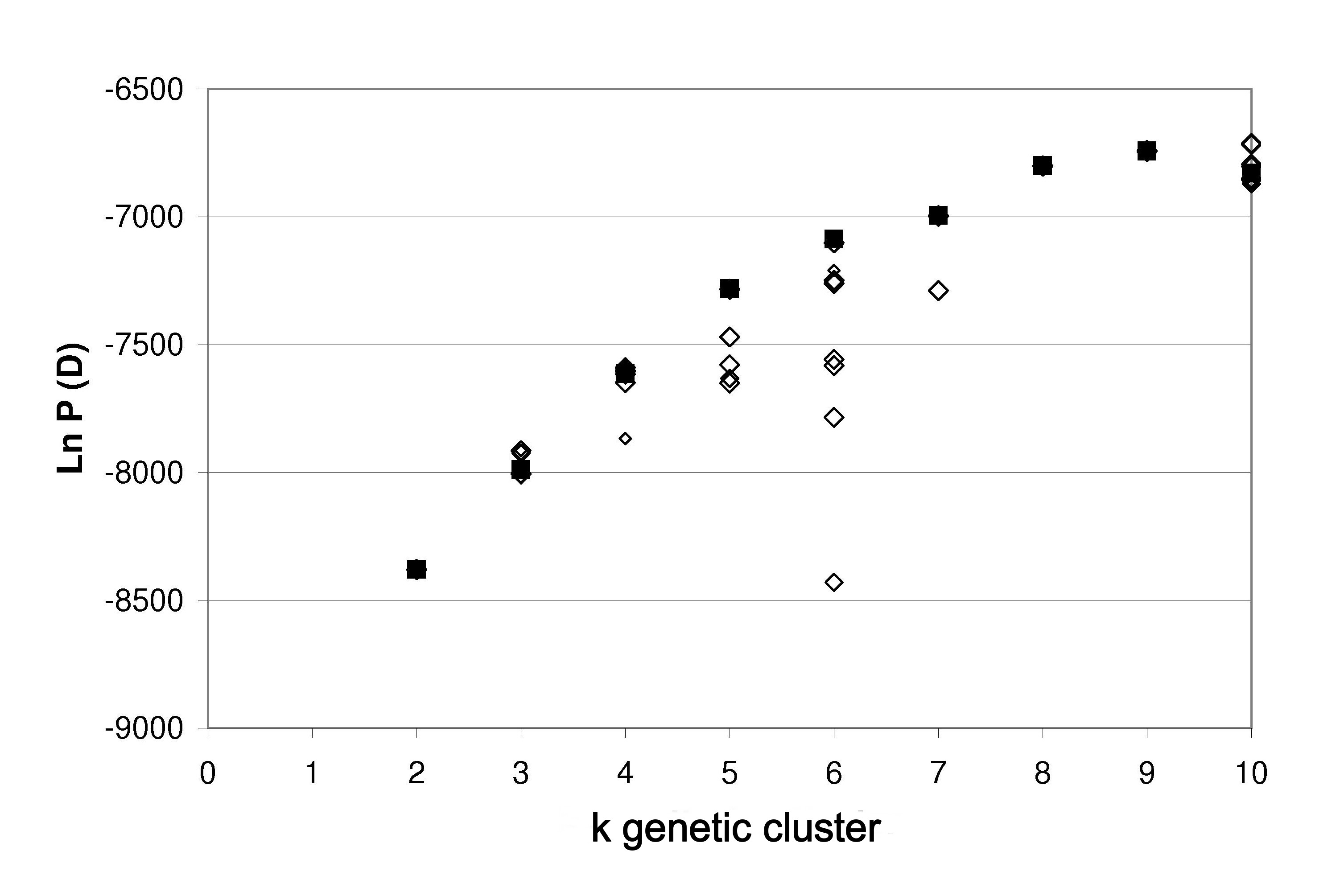

Supplement: Additional file 4 — Distribution of ln(X|K) from ten iterations ranging from K = 2 to K = 10. Values of log likelihood of the multilocus genotypic data, ln(X|K), as a function of the number of clusters, K (ten runs); the largest values of ln(X|K) are presented with black dots. [file 1297-9686-44-5-S4.JPEG]
